# Dr. Eberhard Trams—The man who coined the name “exosomes”—A prescient but largely forgotten pioneer

**DOI:** 10.1002/jev2.12370

**Published:** 2023-10-04

**Authors:** Ewen MacDonald, Norman Salem Jr

**Affiliations:** ^1^ School of Pharmacy (emeritus) University of Eastern Finland Kuopio Finland; ^2^ DSM Nutritional Sciences (retired) Columbia Maryland USA

1

While the approved term to describe the membrane‐bound particles released from cells is now “extracellular vesicles,” there is no doubt that the trivial term “exosomes” is now firmly embedded into the scientific lexicon. Recently, a history of extracellular vesicle research was published (Couch et al., 2021). In it, a passing mention is made to the group working under Dr. Eberhard Trams in the Laboratory of Developmental and Metabolic Neurology in NINCDS, NIH, Bethesda, USA. In a single sentence, it states that Trams et al. in 1981 originally coined the term "exosome" to describe the extracellular vesicles (EVs) shed from the surface of the cell (Trams et al., 1981). As two of the individuals who worked in Trams’ lab in the early 1980s, we would like to explain why this innovative scientist should receive greater recognition—he was one of the first scientists to describe an isolation protocol and then conduct an initial characterization of these extracellular vesicles. The title of the 1981 article is “Exfoliation of membrane ectoenzymes in the form of micro‐vesicles” “but concludes with the words.…such plasma membrane‐derived vesicles could be referred to generically as exosomes” We will describe the tragic circumstances that cut short his pioneering research into extracellular vesicles. To do that we need to go back in time to describe the work being done in that laboratory from about 1979 until 1982.

Like most laboratories in NIH at that time, the Laboratory of Developmental and Metabolic Neurology was just a single room. Dr. Trams had his own table and chair but the other four scientists working in the lab at that time (two Americans, Carl Lauter and Norman Salem Jr. and two Finns, Ewen MacDonald and Matti Reinilä) were crammed into what was essentially a tiny chemical store room. It had only two seats; when all four were present, it was a kind of musical chairs, if a seat became vacant for a few seconds when you went into the laboratory, it would be occupied on your return! During the early 1980s, there were two main projects being conducted in the laboratory—MacDonald and Reinilä were developing assays to measure the different ATPase enzymes present in red blood cell membranes while Lauter and Salem Jr. were working with neural cell cultures—and it is this latter work on which this short historical article will focus.


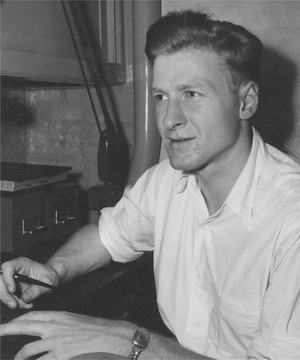


Eberhard Trams at his desk in NIH sometime in the 1970s (photograph courtesy of Penny Trams)

The group (Trams, Lauter, Salem) supplemented by a virologist Ursula Heine, also an NIH researcher, had eventually succeeded in convincing the sceptical reviewers in Biochimica et Biophysica Acta that the preparation of exfoliated vesicles they were describing in their paper was neither an artefact of the isolation procedure nor debris from damaged cells. Trams was frustrated that the article submitted in June 1980 only saw the light of day in July 1981 (Trams et al. 1981). In fact, quite a lot of the article is devoted to supplemental experiments demanded by the reviewers before they would approve its acceptance; it had proved difficult to convince them that the exfoliated micro‐vesicles were truly of physiological significance. Dr. Salem remembers hearing Trams remark to his long‐term colleague, Carl Lauter that many of the criticisms raised by the reviewers were justified and before re‐resubmission, they would have to do some of the supplemental experiments they had proposed. For example, he pondered whether the decision to quantify these structures in terms of mg of protein was really appropriate.

Before looking in more detail at that publication, we would like to give some impressions of Eberhard Trams as a scientist, a section chief and a very unique individual. Trams was born in Germany in 1926. His education was cut short by the Second World War (more about this later) and by the late 1940s he had emigrated to the USA where he continued to study, eventually being awarded a Ph.D. from George Washington University in Washington D.C. In 1958, he moved to work at NIH and in 1976, he was appointed as Chief, Physiology & Metabolism Section, Laboratory of Developmental and Metabolic Neurology. Trams was the archetypical NIH chief, as far as we can remember, he was only away from the laboratory twice in the years 1981–1982; once when he travelled down to the Mote Marine Laboratory in Sarasota, Florida to examine the properties of ATPases in alligators and second when he was part of a team investigating changes in Pacific salmon as they made their final journey up a river in the wilds of British Columbia, Canada to their spawning grounds.

Trams had quite a fiery temper and he certainly did not tolerate any inappropriate behaviour in the laboratory. Despite having lived in the USA for over 30 years, he still had many traces of his German upbringing, for example, his meticulous attention to detail. When approached with a putative new experiment that could be conducted, he would invariably reply “*And how do you intend to interrogate this problem?*” While researching this article, we came across some text in a history of the Mote Marine Laboratory in Florida that perfectly sums up Trams’ approach to science. “*Trams expressed his belief in the importance of scientific fundamentals in this way: Basic research is like a language we must learn. If we have a limited vocabulary, we're just like infants asking the origin of the universe. Most of the time we ask precise questions. We won't get a precise answer unless we ask a precise question.”* (Johnson et al., 1990).

Trams had named the exfoliated vesicles that the group had isolated from cultured cells as “exosomes.” The “exo” part of the word derives from the fact that they were exfoliated and also now extracellular, that is, outside of the cell; the “some” was an indication of how they had been isolated by high‐speed centrifugation and in this respect they resembled two other “somes” recognized at that time, microsomes and synaptosomes. We think that he came up with this term during one of his frequent walks up and down the hallway while of course smoking a cigarette and humming to himself.

When one reads the BBA article through today's eyes, it is amazing how prescient it really was. While much of the text is devoted to the nuts and bolts of the isolation procedure and a characterization of the properties of the exfoliated ATPases shed from cell membranes in vitro as well as the supplemental experiments demanded by the journal's reviewers, it is noteworthy at no place did Trams consider the possibility that these structures were “waste disposal mechanisms.” Instead, he was convinced of their physiological importance. Here are a couple of quotes from the BBA article that sums up his thoughts on their significance. *“The question must be asked if the shedding of microvesicles and their interaction with a target cell or target organ represents a physiologic phenomenon that takes place in vivo?” “It is also conceivable that the vesicle, in part or in toto can be incorporated into a recipient cell thereby producing a modification of the host cell.”* “*The inter‐cellular transport of some trophic substances or nutrients might involve such vesicles as the microvesicles which have been harvested from cell culture superfusates*.” In addition, by comparing the extracellular vesicles to liposomes which had also then recently been postulated to serve as drug delivery vehicles, he speculated that “*the distribution of some cellular products between cells is achieved in a similar way* (with microvesicles as now being attempted with liposomes—our words) *i.e. packaged and provided with an address rather than simply diffused through extracellular fluid compartments.”* The exosomes’ cargo is only briefly described; they did not find any trace of DNA but 5% of the vesicle pellet consisted of RNA. It has to be remembered that Trams’ group specialized in lipid, not protein, biochemistry and in the early 1980s, the identification of proteins was still in its infancy, so beyond the speculation that they might contain trophic substances, there is nothing in the paper about proteins present inside the extracellular vesicles. Dr. Salem well remembers performing the lipid analyses on these quite small biological samples and plasma membrane fractions to demonstrate that they did not simply represent membrane debris. There is also one intriguing glimpse of work underway in the laboratory “*We have recently found that isotopically labelled constituents of the microvesicles can be transferred to recipient cells.”* The initial results of these studies were added to the manuscript in response to one reviewer's questions but further experiments with this approach were planned. Nonetheless, a further elucidation of the interplay between extracellular vesicles and cells was left unpublished and we would like to explain why these crucial experiments were never completed.

In addition to his scientific career, Trams had a great love of small‐plane aviation. He was a founding member of the Congressional Flying Club, which as the name implies, had members from the US Congress and senior administrative figures. There is one amusing anecdote related to his hobby. One day, he was giving a first flying lesson to a senior judicial official. The man remarked to Trams “I hear that you were a fighter pilot in the last war—what plane did you fly? A Mustang?” Trams in his unsmiling deadpan manner replied “No—a Messerschmitt.” According to Trams’ family, their father hated Hitler and all that the Nazi party stood for but nonetheless, he was conscripted into the Luftwaffe and sent to the Eastern Front towards the end of the war. His plane had been shot down by the Russians on his first combat mission but he avoided capture by being hidden by a Romanian physician. After the end of the war, he was back in Berlin as a prisoner of war, charged with clearing rubble from the devastated city. It was there that he met his future wife, Isabelle—a Quaker from Philadelphia. It is hardly surprising that during the time of the Vietnam War, he too had become an avowed pacifist and by the early 1980s he was a supporter of many liberal causes, a position none‐too‐popular in the era of President Reagan.


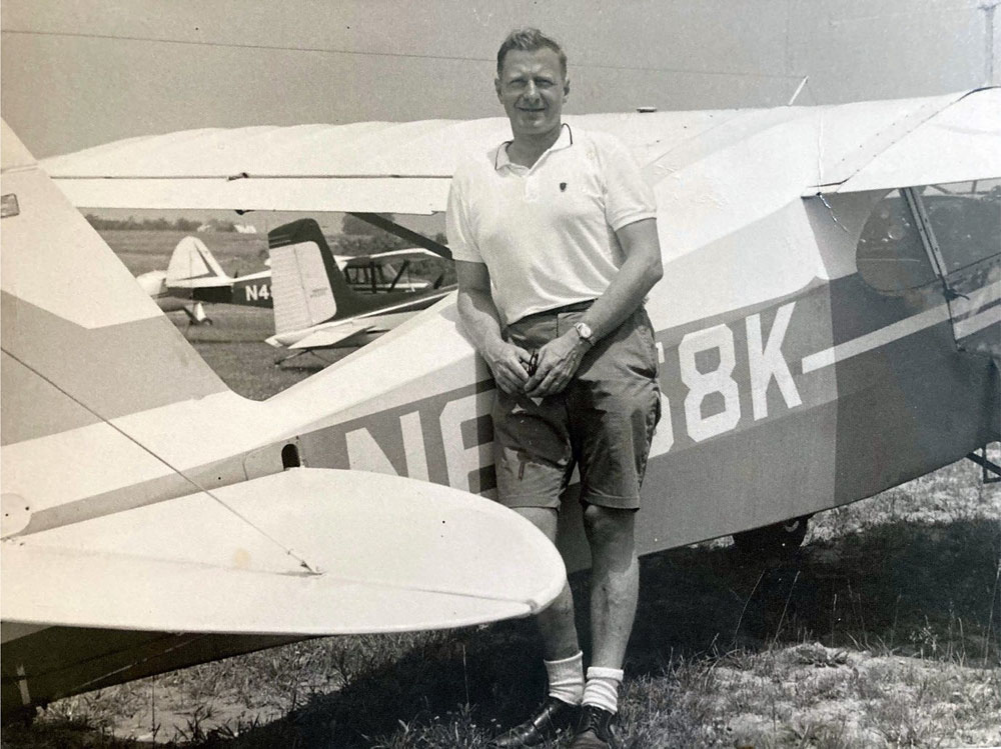


Photograph of Trams with his beloved Cessna airplane (courtesy of Pat Trams)

While Trams was a somewhat intimidating figure to the post‐docs in the lab—after all, he was the boss, our mentor, he was very sociable with his peers in NIH. Thus, it was something of a surprise, when one morning he got up from his table and came across to the part of the lab where the research activities were being done and asked if we had anything that couldn't be interrupted or could the four of us go out for lunch. We went out to the parking lot and he told us to follow his car—what else but a Volkswagen Beetle! We drove out to Gaithersburg, not to a restaurant but to a small airfield and soon we were pushing his small plane out of the hanger. We took off and did some sightseeing over the monuments and buildings in the centre of Washington DC before heading east out to Chesapeake Bay. It was quite alarming when the plane started to descend that there was no runway in sight—just a field. Moments later we had landed in that field and taxied to the back of a small wooden shack. We walked around to find a delightful restaurant right on the Bay and enjoyed soft‐shell crab sandwiches with a cold beer. On the way back, we shared the back seat with a basket of live, in fact very lively, soft‐shell crabs, their hissing almost drowning out the sound of the plane's engine. This was fortunate as the two of us in the back seats were unaware that Trams had handed control of the plane over to Norman Salem, only taking over a few seconds before landing. An hour later, we were back in the laboratory completing the protein assays we had started in the morning. It was moments like these that made working in Trams' lab so memorable.

On March 28^th^ 1982, he took off in his four‐seater Cessna with two passengers on a sightseeing trip. At some point, a warning light came on and he decided to make a landing in the nearest airfield to determine the problem. Since the required part was back at his home base, he accepted a ride in another pilot's plane which was heading that way—it was this plane that crashed, killing all four on board. At his funeral, many of his aviation colleagues remarked that if Trams had been in the pilot's seat, there would never have been a crash, he was too good a pilot.

Later in the week following his death, the Laboratory Chief of NINCDS informed the four of us that without Dr. Trams, the Section would be closed down in a few months and we should all try to finish up as much as possible in that time. The two Finns had already decided to head back to Finland and the two Americans soon found new positions within NIH. It seems probable that in view of the struggle associated with the publication of the BBA article, they decided to wrap up their work with a couple of articles focusing on less controversial topics –and that explains why the unpublished experiments hinted at in the BBA article were never subjected to scientific scrutiny and published. Trams was only 56 when he died, one can only wonder at how his pioneering work on extracellular vesicles would have progressed in the next decades but for that fateful flight at the end of March 1982.

## AUTHOR CONTRIBUTIONS

**Ewen MacDonald**: Writing—original draft; writing—review and editing. **Norman Salem**: Writing—original draft; writing—review and editing.

## CONFLICTS OF INTEREST STATEMENT

Neither Ewen MacDonald nor Norman Salem Jr have any conflicts of interest with respect to this article
